# Availability and inequality in accessibility of health centre-based primary healthcare in Ethiopia

**DOI:** 10.1371/journal.pone.0213896

**Published:** 2019-03-29

**Authors:** Abraha Woldemichael, Amirhossein Takian, Ali Akbari Sari, Alireza Olyaeemanesh

**Affiliations:** 1 Department of Health Management and Economics, School of Public Health, Tehran University of Medical Sciences, Tehran, Iran; 2 School of Public Health, College of Health Sciences, Mekelle University, Mekelle, Ethiopia; 3 Department of Global Health and Public Policy, School of Public Health, Tehran University of Medical Sciences, Tehran, Iran; 4 Health Equity Research Centre (HERC), Tehran University of Medical Sciences, Tehran, Iran; 5 National Institute for Health Research, Tehran, Iran; Jawaharlal Nehru University, INDIA

## Abstract

**Background:**

Achieving fair access to healthcare and improving population health are crucial in all settings. Properly staffed and fairly distributed primary health care (PHC) facilities are prerequisites to ensure accessible healthcare services. Nevertheless, availability and accessibility issues are common public health concerns, especially in under-resourced countries including Ethiopia. Measuring inequalities in accessibility of healthcare resources guide policy decisions to improve PHC services and ultimately achieving universal health coverage (UHC).

**Purpose:**

To assess availability and measure magnitude and trend of inequalities in accessibility of health centre-based PHC resources in Ethiopia during 2015 to 2017.

**Methods:**

We conducted a cross-sectional population-based analysis of district-level data collected from 16^th^ December 2017 until 24^th^ May 2018. Afar, Dire-Dawa, and Tigray regions were purposefully included in the study to represent the four pastoralist/semi-pastoralist, three urban and four agrarian regions in Ethiopia, respectively. We used ratios, different inequality indices and Gini decomposition techniques to characterise the inequalities.

**Results:**

In 2017, median of health centres (HCs) per 15,000 inhabitants and their Gini indices (GIs) for Afar, Dire-Dawa, and Tigray were 0.781, 0.566, 0.591 vs. 0.237, 0.280, 0.216 respectively. Median overall skilled health workers (SHWs) per 10,000 inhabitants were 5.250, 7.539, and 6.246, respectively. These accounted for 11.80%, 16.94% and 14.04% of the WHO target of 44.5 to achieve SDGs. The corresponding GIs for the regions were 0.347, 0.186 and 0.175. Despite a higher overall SHWs inequality in the urban districts of Tigray (GI = 0.301), only Tigray showed significant inequality reductions in GHE (p < 0.001) and in all categories of SHWs (p < 0.05).

**Conclusions:**

Our analysis provided a clear picture of availability and inequalities in PHC resources across three regions in Ethiopia. Identifying contributing factors to low densities and high inequalities of SHWs may help improve PHC services nationwide, along with pathway towards UHC.

## Introduction

Achieving fair access to healthcare and improving population health are crucial in all settings [1,2]. Proponents of the human rights hold that healthcare (both public and clinical) is a human right, which is practically applied and has a strong moral foundation [3,4]. The rights-based approach to healthcare is believed to improving people’s access, especially in low- and middle income countries [5]. Nevertheless, as healthcare is a scarce resource that must be rationed, identifying the root problems of unaffordable healthcare is essential to fix appropriate solutions accordingly [6]. Others hold that the right to health and healthcare should balance both the social interests and individual rights. Hence, whether healthcare is a human right or not is a continuing debate [7].

Access is a complex concept. Access to healthcare is understood as a means to realising objectives such as greater efficiency and better-quality services. Despite the complex processes involved, the universal health coverage (UHC) plays a vital role in accessing the healthcare services for all. The sustainable development goals (SDGs) do not consider the UHC target as an end itself. Rather, UHC is a political choice to strengthen countries’ ever-changing complex systems towards sustainable health development [8,9].

The difference in viewpoints concerning access and access to healthcare is a continuing debate. Hence, there is no single method to measure access to healthcare. Despite various views towards access, i.e., political, an opportunity to use healthcare, the first step towards health for all, and use and availability of healthcare resources, none of which defines access comprehensively. Access is a general concept that represents the degree of fit between the clients and the healthcare system. The set of specific, independent and interconnected dimensions that help optimise and measure access to healthcare are availability, accessibility, accommodation, affordability, and acceptability [10].

Ethiopia is one of the least developing countries in the world and the second populous country in Africa. The Ethiopian government has been committed to improving the accessibility of essential healthcare services to all citizens through decentralisation, encouraging partnership with the private sector and inclusive participation of stakeholders. The healthcare has been reoriented to achieve the universal access to PHC by the year 2017 and improve the responsiveness of healthcare to people's needs and expectations [11]. Nevertheless, the weak infrastructure, limited distribution systems, and the poor services quality have hindered universal access to healthcare services [12]. Our study focused on the availability and accessibility dimensions of access.

Accessibility refers to the spatial and people aspects of healthcare services and is subject to adequacy of available healthcare resources and their fair distributions. Thus, the accessibility of the healthcare resources and services can be measured by using either the place or people-based approach. The place-based approach requires pertinent information concerning the spatial or physical proximity between the point of service production and the stable residences of the service users. The second approach considers the service users’ characteristics including their culture, life style, time and other resources to use the services [10,13,14].

Despite the extensive search, we found limited evidence concerning the district-based availability, adequacy, and fairness in the distributions of PHC resources and services in Ethiopia. This study aims to assess the provider perspective of people-based availability and accessibility of healthcare services in three representative regions (Afar, Dire-Dawa, and Tigray) in Ethiopia from 2015 to 2017. The findings are anticipated to contribute to the reduction of the provider perspective barriers to healthcare and improvements in PHC services and ultimately achieving health-related SDGs across Ethiopia and perhaps beyond.

## Methods

### Study design and settings

We used a cross-sectional study to analyse the magnitude and trend of inequalities in the accessibility of health centre-based PHC resources of districts in three regions in Ethiopia. Afar, Dire-Dawa and Tigray regions were purposefully included in the study to represent the four pastoralist/agro-pastoralist, three urban, and four agrarian regions in Ethiopia, respectively. Tigray is located in northern Ethiopia and consist of 52 districts (18 urban or municipalities and 34 rural districts). Afar stretches from Awash (near the centre) to the northeast (bordering Djibouti and Eritrea), and has 34 districts (two urban or municipalities and 32 rural). Dire-Dawa is located in eastern Ethiopia, and has only one administrative district consisting of nine health centres (HCs) catchment areas (six in urban and three in rural settings) or “operational districts” henceforth districts.

### Data sources and data collection

We used the Central Statistical Agency (CSA) of Ethiopia’s census-based annual population estimates, annual total government health expenditure (GHE) in Ethiopian Birr, the total number of functional HCs and SHWs (health officers, nurses, midwives, and their cumulative summation) who provide services within HCs in each district. Data were collected from 16^th^ December 2017 until 24^th^ May 2018 using a tailored template.

The population estimates of the districts were obtained from the Planning and Policy Directorate of the Federal Ministry of Health of Ethiopia (FMOH). The annual approved total GHE for each district and the total number of functional HCs in the districts were obtained from the Bureau of Finance and Economic Development (BoFED), and Bureau of Health in each region respectively. The total number of SHWs by professional category were obtained from the Human Resources Management (HRM), and Planning and Budgeting directorates at the Regional Health Bureaus (RHBs), and from the HRM of the Woreda (district) Health Offices in the regions.

### Analysis and interpretation

Our analysis was based on data from 51 districts (17 urban or municipalities and 34 rural districts) in Tigray, nine operational districts (six urban and three rural) in Dire-Dawa and 34 districts (two urban or municipalities and 32 rural districts) in Afar region. One urban district in Tigray was excluded from the analysis because it did not have health centre-based PHC services provision during the study period (S1 Dataset). First, we calculated the annual GHE per capita in Birr and the ratios of the HCs per 15,000 inhabitants of each district. Besides, the district-level annual ratios of the SHWs were calculated per HC, and per 10,000 inhabitants separately [15].

We then applied the people-based accessibility of healthcare services approach [13] in the egalitarianism perspective, which assumes equal accessibility of essential healthcare services to all regardless of cost [14,16]. The unit for the analysis was district. We compared the densities and the accessibilities of the healthcare resources between urban and rural districts in Tigray and Dire-Dawa, and among categories of districts in Afar region, taking into account the hardship allowance (HA) and average altitudes of each district in metres above sea level (masl). We used mean with standard deviation (SD) and median with interquartile range (IQR) to describe the distributions during the year 2017.

The Theil, Atkinson, and the Gini indices were used to describe the inequalities. We applied the Theil T (TT) and Theil L (Mean Logarithmic Deviation (MLD)) measures. The Theil T was performed at α = 1, while the Theil L was computed at α = 0 [17]. The TT shows changes that affect the upper tail of the distribution and the MLD is sensitive to changes that affect the lower tail of the distribution. The values fall in the interval [0, ∞) and the value closer to zero signifies more equality and higher value more inequality [18,19].

Furthermore, we analysed the Atkinson indices (AIs) at the inequality aversion parameter (epsilon) of 0.5, 1, and 2. The higher the epsilon (ε) value, the more sensitive the Atkinson index becomes to inequalities at the bottom of a distribution. We further calculated the Gini index (GI), which is a sensitive indicator to small changes in the mean and location of the distributions. The Atkinson and Gini values fall in the interval [0,1], where the values closer to zero show more equality and higher values more inequality [18,20]. We performed the overall Gini value decomposition of each SHWs category during 2017 into three components: net between district Gini (**G**_**B**_), the interaction term Gini (**G**_**I**_) and the net within district Gini (**G**_**W**_). These Gini components additively provide the overall Gini value of a distribution. The interaction term is an overlap in the distributions between the categories for the districts. This technique clearly shows the net between-group difference by addressing the issues of the reranking effect [21]. The change in inequality between 2015 and 2017 for the distributions was evaluated using the Gini decomposition at two points in time [22]. We used bootstrap technique to determine the 95% confidence interval (CI) for the indices. STATA version 14.1 was used for the analysis and considered significant at a p-value of 0.05. The discussions of the findings were based on the Gini values.

## Results

### Government health expenditure (GHE)

The mean and median of GHE per capita and Gini value for each region is presented in Table 1. The median difference (β) in GHE per capita in Tigray (β = -18.057 Birr, 95%CI: -42.598–6.484; p = 0.146) and in Dire-Dawa (β = 60.452 Birr, 95%CI: -71.984–192.887; p = 0.316) between the urban and rural residents were statistically insignificant. In Afar, the median GHE per capita for the residents of no hardship allowance (NHA) districts (Med. = 236.881Birr, IQR: 154.731–287.926) was higher than those residents in the districts with HA (Med. = 149.629 Birr, IQR: 119.274–206.714) but the difference was statistically insignificant (β = 83.995, 95%CI: -15.733–183.722; p = 0.096). Besides, a reduction in the GHE per capita was observed as the altitudes of the districts decline. The GI of the GHE per capita was the lowest for Dire-Dawa (GI = 0.152) and the highest for Afar (GI = 0.268).

**Table 1:** Government health expenditure and health centres distributions across three regions and their Gini values in 2017.

Indicator/Region		District	No.*+	Mean (SD)	Median (IQR)	Gini Index, (95%CI)
**GHE per capita**						
	Tigray	**Total**	**51**	**141.364 (62.293)**	**128.457 (109.873, 151.642)**	**0.181 (0.118, 0.244)**
		Urban	17	142.867 (49.466)	144.299 (103.113, 178.420)	0.192 [0.143, 0.241)
		Rural	34	140.613 (68.493)	125.922 (114.111, 141.081)	0.163 (0.068, 0.257)
	Dire-Dawa	**Total**	**9**	**252.003 (76.664)**	**279.962 (240.473, 292.663)**	**0.152 [0.064, 0.239]**
		Urban	6	228.501 (79.875)	271.715 (136.020, 282.123)	0.162 [0.052, 0.272]
		Rural	3	299.007 (50.879)	323.920 (240.473, 332.628)	0.068 [0.004, 0.132]
	Afar	**Total**	**34**	**222.931 (114.058)**	**198.380 (139.588, 277.452)**	**0.268 [0.207, 0.329)**
	Allowance	NHA	20	246.423 (118.016)	236.881 (154.731, 287.926)	0.245 [0.175, 0.314]
		30%	10	201.888 (117.436)	149.629 [132.931, 305.514]	0.296 [0.193, 0.399]
		40%	4	158.077 (52.495)	157.565 (112.701, 203.453]	0.148 [0.046, 0.249]
	Altitude (masl)	>1000	3	279.132 (132.931)	256.647 (158.878, 421.871)	0.209 (0.061, 0.358]
		500–1000	19	253.176 (119.160)	242.872 (145.279, 305.514)	0.242 [0.164, 0.320]
		< 500	12	160.993 (77.157)	136.260 (113.711, 201.641)	0.239 [0.153, 0.326)
**HC/15000 pop**						
	Tigray	**Total**	**216**	**0.626 (0.324)**	**0.591 (0.434, 0.730)**	**0.216 (0.133, 0.300]**
		Urban	28	0.478 (0.167)	0.426 (0.374, 0.585)	0.192 [0.146, 0.239)
		Rural	188	0.699 (0.358)	0.624 (0.534, 0.740)	0.200 (0.094, 0.306]
	Dire-Dawa	**Total**	**15**	**0.598 (0.334)**	**0.566 [0.475, 0.595]**	**0.280 [0.168, 0.391]**
		Urban	9	0.422 (0.163)	0.479 [0.218, 0.566)	0.186 (0.070, 0.302)
		Rural	6	0.951 (0.316)	1.057 [0.595, 1.200]	0.141 [0.024, 0.259]
	Afar	**Total**	**92**	**0.857 (0.422)**	**0.781 (0.562, 1.062)**	**0.237 [0.164, 0.311)**
	Allowance	NHA	54	0.948 (0.465)	0.837 (0.650, 1.144)	0.223 [0.128, 0.317)
		30%	24	0.693 (0.350)	0.565 (0.479, 1.012)	0.261 (0.165, 0.357)
		40%	14	0.814 (0.266)	0.727 (0.628, 1.001)	0.147 [0.059, 0.234]
	Altitude (masl)	>1000	7	0.769 (0.219)	0.709 (0.586, 1.012)	0.123 (0.045, 0.202)
		500–1000	54	1.006 (0.471)	0.907 (0.714, 1.205)	0.218 [0.106, 0.330]
		< 500	31	0.644 (0.268)	0.581 (0.503, 0.735)	0.213 [0.119, 0.306)

* Values under “No.” for government health expenditure (GHE) per capita refer to number of districts

+: Values under “No.” for the HC/15,000 population refer to number of health centres; masl: metres above sea level; NHA: no hardship allowance

### Availability of health centres

The overall densities of HCs in Tigray, Dire-Dawa and Afar regions were 0.591, 0.566 and 0.781 per 15,000 inhabitants during 2017 respectively (Table 1). The median difference of HCs between the rural and urban districts in Tigray (β = 0.199, 95%CI: 0.080–0.317; p = 0.001) and in Dire-Dawa (β = 0.582, 95%CI: 0.189–0.975; p = 0.010) were statistically significant. In Afar, the median difference (β = 0.220, 95%CI: -0.136–0.575; p = 0.217) between the NHA districts (Med. = 0.837, IQR: 0.650–1.144) and districts with HA (Med. = 0.628, IQR: 0.528–1.012) was insignificant. The Gini value of the HCs was relatively higher for Dire-Dawa (GI = 0.280) than that of the other two regions.

### Availability and density of SHWs

The densities of the different categories of SHWs in each region is presented in Table 2. The median SHWs per HC was lowest for HOs in Afar (Med. = 1.417, IQR: 1.000–2.000) and highest for nurses in Dire-Dawa (Med. = 14.000, IQR: 13.000–14.000). The median overall SHWs per 10,000 inhabitants in Afar, Tigray, and Dire-Dawa were 5.250 (IQR: 3.580–8.488), 6.246 (IQR: 5.007–7.293), and 7.539 (IQR: 6.435–8.228) respectively. In Tigray, the median difference between the urban and rural districts for HOs (β = 0.047, 95%CI: -0.321–0.415; p = 0.800), nurses (β = -0.551, 95%CI: -1.450–0.348; p = 0.224), midwives (β = -0.078, 95%CI: -0.411–0.254; p = 0.637), and overall SHWs (β = -0.925, 95%CI: -2.154–0.304; p = 0.137) were statistically insignificant. In Dire-Dawa, the density of midwives in the rural districts was significantly higher than in the urban districts (β = 1.165, 95%CI: 0.294–2.035; p = 0.016). The overall SHWs density in the rural districts (Med. = 10.217, IQR: 7.539–10.800) was higher than in the urban districts (Med. = 6.939, IQR: 4.058–7.543), but the difference was statistically insignificant (β = 3.783, 95%CI: -0.447–8.014; p = 0.072). In Afar, the density of HOs in the NHA districts (Med. = 1.167, IQR: 0.548–1.878) was significantly higher (β = 0.780, 95CI: 0.042–1.517; p = 0.039) than in districts with HA (Med. = 0.428, IQR: 0.352–0.911). The overall SHWs density in the NHA districts (Med. = 8.233, IQR: 3.950–10.272) was also significantly higher (β = 4.396, 95%CI: 0.388–8.404; p = 0.033) than in the districts with HA (Med. = 3.833, IQR: 2.517–4.834).

**Table 2:** Average densities of SHWs categories per health centre and per 10,000 inhabitants by region and district category during 2017.

SHWs/Region		Context	No.	SHWs per health centre		SHWs per 10000 inhabitants	
				Mean (SD)	Median (IQR)	Mean (SD)	Median (IQR)
**Tigray**							
	HOs	**Total**	**577**	**3.002 (1.243)**	**2.750 (2.167, 3.500)**	**1.182 (0.552)**	**1.035 (0.811, 1.364)**
		Urban	110	3.971 (1.626)	4.000 (3.000, 5.000)	1.271 (0.703)	1.035 (0.779, 1.892)
		Rural	467	2.517 (0.578)	2.563 (2.000, 3.000)	1.138 (0.465)	1.058 (0.879, 1.293)
	Nurses	**Total**	**2017**	**10.505 (3.781)**	**9.600 (7.750, 13.000)**	**4.055 (1.470)**	**4.101 (3.018, 4.732)**
		Urban	389	14.108 (3.930)	13.000 (12.000, 16.000)	4.353 (1.459)	4.328 (3.783, 5.519)
		Rural	1628	8.703 (2.036)	8.586 (7.375, 9.667)	3.906 (1.475)	3.769 (3.018, 4.519)
	Midwives	**Total**	**650**	**3.224 (0.817)**	**3.000 (2.714, 3.833)**	**1.270 (0.432)**	**1.340 (0.897, 1.481)**
		Urban	107	3.912 (0.797)	4.000 (3.333, 4.500)	1.235 (0.428)	1.364 (0.923, 1.455)
		Rural	543	2.880 (0.581)	3.000 (2.500, 3.167)	1.288 (0.440)	1.247 (0.897, 1.506)
**Dire-Dawa**							
	HOs	**Total**	**41**	**3.778 (3.183)**	**3.000 (2.000, 3.000)**	**0.991 (0.370)**	**0.965 (0.794,1.200)**
		Urban	30	3.250 (3.574)	3.250 (3.000, 3.500)	0.919 (0.401)	0.957 (0.507, 1.145)
		Rural	11	1.833 (0.289)	2.000 (1.500, 2.000)	1.134 (0.313)	1.200 (0.794, 1.409)
	Nurses	**Total**	**206**	**14.111 (3.847)**	**14.000 (13.000, 14.000)**	**5.030 (1.704)**	**5.154 (4.503, 6.328)**
		Urban	141	15.750 (3.313)	14.000 (13.500, 20.000)	4.304 (1.570)	4.703 (2.900, 5.154)
		Rural	65	10.833 (2.754)	9.500 (9.000, 14.000)	6.483 (0.842)	6.694 (5.555, 7.200)
	Midwives	**Total**	**47**	**3.056 (0.635)**	**3.000 (3.000, 3.000)**	**1.204 (0.664)**	**1.131 (0.949, 1.190)**
		Urban	29	3.083 (0.801)	3.000 (3.000, 3.000)	0.856 (0.329)	0.957 (0.652, 1.131)
		Rural	18	3.000 (0.000)	3.000 (3.000, 3.000)	1.901 (0.632)	2.114 (1.190, 2.400)
**Afar**							
	HOs	**Total**	**160**	**1.887 (1.361)**	**1.417 (1.000, 2.000)**	**1.096 (0.897)**	**0.718 (0.392, 1.749)**
		NHA	108	2.221 (1.655)	1.584 (1.000, 2.584)	1.357 (1.005)	1.167 (0.548, 1.878)
	Allowance	30%	34	1.433 (0.498)	1.167 (1.000, 2.000)	0.683 (0.528)	0.409 (0.352, 0.911)
		40%	18	1.354 (0.695)	1.167 (0.875, 1.833)	0.827 (0.706)	0.575 (0.368, 1.287)
	Altitude	>1000	15	2.667 (2.082)	2.000 (1.000, 5.000)	1.356 (0.904)	1.799 (0.315, 1.952)
	(masl)	500–1000	105	2.079 (1.555)	1.500 (1.000, 2.500)	1.353 (1.001)	1.124 (0.562, 1.874)
		< 500	40	1.389 (0.560)	1.167 (1.000, 2.000)	0.625 (0.498)	0.428 (0.328, 0.653)
	**Nurses**	**Total**	**667**	**7.282 (3.613)**	**7.000 (4.333, 9.333)**	**4.274 (3.150)**	**3.132 (2.062, 6.069)**
	Allowance	NHA	425	8.058 (3.863)	7.125 (4.833, 10.333)	5.109 (3.437)	4.813 (2.587, 6.568)
		30%	184	7.100 (2.744)	7.000 (6.000, 9.333)	3.580 (2.537)	2.986 (1.769, 4.951)
		40%	58	3.854 (2.593)	3.250 (1.833, 5.875)	1.838 (0.909)	1.577 (1.202, 2.474)
	Altitude (masl)	>1000	66	10.00 (3.180)	11.667 (6.333, 12.000)	5.183 (2.477)	4.685 (2.993, 7.871)
		500–1000	402	7.425 (3.311)	7.000 (4.333, 9.333)	5.102 (3.489)	4.941 (2.507, 7.024)
		< 500	199	6.375 (4.054)	6.500 (3.250, 7.250)	2.738 (2.177)	2.180 (1.498, 3.049)
	**Midwives**	**Total**	**215**	**2.507 (1.519)**	**2.417 (1.667, 3.000)**	**1.429 (1.020)**	**1.159 (0.719, 2.125)**
	Allowance	NHA	148	2.946 (1.614)	2.583 (1.833, 3.500)	1.789 (1.038)	1.375 (0.999, 2.378)
		30%	58	2.367 (0.958)	2.583 (2.000, 3.000)	1.138 (0.786)	0.984 (0.403, 2.122)
		40%	9	0.667 (0.495)	0.542 (0.292, 1.042)	0.352 (0.262)	0.294 (0.183–0.521)
	Altitude (masl)	>1000	22	1.858 (0.137)	1.846 (1.727, 2.000)	1.690 (0.507)	1.562 (1.260, 2.249)
		500–1000	133	1.734 (0.593)	1.769 (1.111, 2.267)	1.700 (1.007)	1.333 (0.899, 2.286)
		< 500	60	1.998 (1.450)	1.474 (1.077, 2.659)	0.933 (0.999)	0.616 (0.280, 1.129)

**HOs:** health officers; **masl**: metres above sea level; **NHA**: no hardship allowance; **SHWs**: skilled health workers

### Inequality in accessibility of SHWs

Table 3 presents the Gini values of each category of SHWs per 10,000 inhabitants from 2015 to 2017 and the decomposition of the overall Gini values for 2017. Tigray and Dire-Dawa had lower Gini values for all the distributions than the values for the Afar. The net within district difference in SHWs in Tigray and Afar, and between district differences except for the HOs in Dire-Dawa dominantly explained the overall inequalities in each category of the SHWs. The net within district difference in the overall SHWs in Tigray and Afar accounted for 53.74% and 47.64% of the overall inequality in the cumulative SHWs of each region respectively. However, the net between district difference in the overall SHWs in Dire-Dawa explained 57.02% of the overall inequality of the aggregate SHWs in the region.

**Table 3:** Gini values for accessibility of SHWs from 2015–2017 and overall Gini value components in 2017.

Region/SHW			Gini index (95%CI)			Overall Gini components (GI, %)	
			Year 2015	Year 2016	Year 2017		
**Tigray**							
	HOs	**Total**	0.302 (0.244, 0.360]	0.246 (0.196, 0.296]	**0.246 (0.198, 0.294]**	**G**_**B**_	0.025 (10.148)
		Urban	0.266 (0.170, 0.361]	0.287 (0.193, 0.381)	0.301 (0.216, 0.387)	**G**_**I**_	0.099 (40.348)
		Rural	0.269 (0.205, 0.333)	0.191 [0.137, 0.245)	0.201 [0.138, 0.263)	**G**_**W**_	**0.122 (49.504)**
	Nurses	**Total**	0.230 [0.185, 0.275]	0.187 [0.138, 0.235]	**0.193 (0.151**, **0.234]**	**G**_**B**_	0.025 (12.736)
		Urban	0.197 (0.134, 0.260]	0.189 [0.134, 0.245)	0.179 (0.114, 0.245)	**G**_**I**_	0.066 (34.287)
		Rural	0.213 [0.141, 0.284]	0.179 (0.113, 0.245]	0.188 (0.134, 0.243)	**G**_**W**_	**0.102 (52.977)**
	Midwives	**Total**	0.217 (0.186, 0.248)	0.180 (0.150, 0.210]	**0.187 [0.149**, **0.225]**	**G**_**B**_	0.009 (04.946)
		Urban	0.205 (0.153, 0.258)	0.153 [0.096, 0.210)	0.185 [0.112, 0.257)	**G**_**I**_	0.075 (39.929)
		Rural	0.216 (0.173, 0.260]	0.190 (0.157, 0.224)	0.185 (0.145, 0.225]	**G**_**W**_	**0.103 (55.126)**
							
	Overall	**Total**	0.217 (0.177, 0.248)	0.169 [0.134, 0.204]	**0.175 (0.137, 0.214)**	**G**_**B**_	0.018(10.284)
	SHWs	Urban	0.217 (0.177, 0.257]	0.172 [0.108, 0.235)	0.170 [0.110, 0.230)	**G**_**I**_	0.063 (35.979)
		Rural	0.202 (0.144, 0.259)	0.159 [0.103, 0.215]	0.172 (0.124, 0.219]	**G**_**W**_	**0.094 (53.736)**
							
**Dire-Dawa**							
	HOs	**Total**	0.230 (0.138, 0.322]	0.230 (0.137, 0.323)	**0.199 (0.114, 0.295]**	**G**_**B**_	0.048 (24.248)
		Urban	0.226 [0.115, 0.336)	0.220 (0.116, 0.325]	0.220 [0.121, 0.320)	**G**_**I**_	0.045 (22.513)
		Rural	0.155 [0.014, 0.297)	0.192 [0.019, 0.365]	0.121 (0.033, 0.208]	**G**_**W**_	**0.106 (53.239)**
	Nurses	**Total**	0.182 [0.085, 0.280)	0.179 [0.089, 0.269]	**0.178 [0.085, 0.272)**	**G**_**B**_	**0.096 (53.969)**
		Urban	0.185 [0.083, 0.287]	0.185 (0.080, 0.289]	0.185 (0.083, 0.287)	**G**_**I**_	0.004 (2.127)
		Rural	0.119 (0.032, 0.207)	0.079 [0.016, 0.142]	0.056 [0.018, 0.095]	**G**_**W**_	0.078 (43.904)
	Midwives	**Total**	0.252 [0.145, 0.259]	0.276 (0.152, 0.401)	**0.276 (0.137**, **0.416]**	**G**_**B**_	**0.193 (69.800)**
		Urban	0.176 (0.070, 0.283)	0.186 (0.037, 0.335)	0.186 (0.052, 0.320)	**G**_**I**_	-0.000 (-0.000)
		Rural	0.116 (0.012, 0.220]	0.141 (0.026, 0.257)	0.141 [0.026, 0.257)	**G**_**W**_	0.083 (30.200)
							
	Overall	**Total**	0.193 [0.089, 0.296)	0.188 [0.091, 0.285)	**0.186 [0.089, 0.296)**	**G**_**B**_	**0.106 (57.023)**
	SHWs	Urban	0.178 (0.063, 0.293]	0.177 (0.064, 0.290]	0.177 [0.074, 0.280]	**G**_**I**_	0.002 (1.272)
		Rural	0.124 (0.040, 0.209)	0.087 [0.005, 0.168]	0.076 (0.015, 0.138)	**G**_**W**_	0.077 (41.706)
							
**Afar**							
	HOs	**Total**	0.386 (0.303, 0.469]	0.348 (0.290, 0.406)	**0.412 (0.341, 0.484)**	**G**_**B**_	0.140 (33.900)
		NHA	0.347 [0.237, 0.458)	0.309 (0.234, 0.384)	0.381 [0.278, 0.484)	**G**_**I**_	0.067 (16.348)
		HA	0.291 (0.207, 0.376]	0.360 [0.265, 0.454]	0.375 [0.286, 0.464]	**G**_**W**_	**0.205 (49.752)**
	Nurses	**Total**	0.352 (0.284, 0.420]	0.365 [0.279, 0.450)	**0.372 [0.288, 0.456)**	**G**_**B**_	0.115 (30.863)
		NHA	0.296 [0.223, 0.368]	0.325 (0.219, 0.431)	0.332 (0.224, 0.441)	**G**_**I**_	0.073 (19.662)
		HA	0.397 [0.266, 0.528)	0.372 [0.274, 0.470)	0.382 [0.278, 0.486]	**G**_**W**_	**0.184 (49.475)**
	Midwives	**Total**	0.391 [0.305, 0.476)	0.369 [0.295, 0.442)	**0.385 [0.315, 0.455)**	**G**_**B**_	0.148 (38.558)
		NHA	0.342 (0.243, 0.441]	0.311 [0.236, 0.386)	0.310 [0.245, 0.374]	**G**_**I**_	0.055 (14.296)
		HA	0.410 [0.305, 0.517)	0.406 (0.301, 0.512]	0.436 [0.325, 0.547]	**G**_**W**_	**0.182 (47.146)**
							
	Overall	**Total**	0.339 [0.281, 0.397)	0.334 [0.271, 0.396)	**0.347 [0.275, 0.419)**	**G**_**B**_	0.126 (36.294)
	SHWs	NHA	0.281 (0.214, 0.349]	0.288 (0.212, 0.365]	0.295 [0.205, 0.384]	**G**_**I**_	0.056 (16.070)
		HA	0.364 [0.243, 0.486)	0.341 (0.230, 0.451]	0.352 (0.253, 0.451]	**G**_**W**_	**0.165 (47.635)**

**HA**: hardship allowance; **HOs:** health officers; **NHA**: no hardship allowance; **SHWs**: skilled health workers

Our analysis also revealed that the Atkinson index at epsilon value of two (ε = 2) and the GI were more sensitive to the inequalities of almost all the distributions analysed. The indices for each category of the SHWs and the overall SHWs in Tigray and Dire-Dawa (Table 4) were lower than the indices for Afar in 2017 (Table 5). The AI at ε = 2 and the GI for Tigray showed higher overall SHWs inequality in the urban districts (AI = 0.396; GI = 0.301) than in the rural districts. The AI at ε = 2 and the GI for the overall midwives in Dire-Dawa (AI = 0.291; GI = 0.276) revealed relatively higher inequality than the other categories of SHWs in the region. In Afar, all the indices except the AI at ε = 0.5 indicated high inequality for midwives working in districts with altitudes of less than 500 masl. The indices revealed that there was high overall SHWs inequality in districts with 30% HA than in the other categories of districts.

**Table 4:** Inequality indices for accessibility of SHWs in Tigray and Dire-Dawa regions during 2017.

Region/SHWs			Theil Indices (95%CI)		Atkinson Indices (95%CI)			Gini Index (95%CI)
			TT at α = 1	TL at α = 0	AI at ε = 0.5	AI at ε = 1	AI at ε = 2	
**Tigray**								
	HOs	**Total**	**0.101 [0.067, 0.135]**	**0.110 [0.063, 0.157)**	**0.051 (0.033, 0.069]**	**0.104 (0.063, 0.146]**	**0.235 [0.108, 0.361]**	**0.246 (0.208, 0.285)**
		Urban	0.071 [0.029, 0.113)	0.069 [0.031, 0.106)	0.034 [0.015, 0.053]	0.066 [0.031, 0.101]	0.127 (0.066, 0.188]	0.201 [0.144, 0.257]
		Rural	0.151 [0.061, 0.241)	0.189 (0.049, 0.330)	0.080 (0.030, 0.131]	0.172 [0.058, 0.287]	0.396 [0.137, 0.655)	0.301 [0.214, 0.388]
	Nurses	**Total**	**0.061[0.033, 0.089)**	**0.061 (0.036, 0.087)**	**0.030 [0.017, 0.043)**	**0.059 (0.036, 0.083]**	**0.116 (0.075, 0.156]**	**0.193 (0.149, 0.237)**
		Urban	0.056 [0.021, 0.091]	0.063 (0.027, 0.099]	0.029 (0.012, 0.047)	0.061 (0.027, 0.095]	0.128 (0.065, 0.192)	0.179 (0.112, 0.246]
		Rural	0.061 (0.022, 0.101]	0.059 (0.024, 0.093]	0.030 [0.011, 0.048)	0.057 [0.024, 0.090)	0.107 (0.053, 0.160]	0.188 [0.129, 0.248)
	Midwives	**Total**	**0.056 (0.038, 0.075)**	**0.059 (0.040, 0.078]**	**0.028 [0.019, 0.038)**	**0.057 [0.039, 0.075]**	**0.115 (0.080, 0.150)**	**0.187 [0.153, 0.222)**
		Urban	0.061 (0.023, 0.099)	0.069 (0.029, 0.110)	0.032 (0.013, 0.051]	0.067 (0.029, 0.105)	0.142 [0.071, 0.213]	0.185 [0.111, 0.258)
		Rural	0.054 (0.033, 0.075]	0.053 (0.033, 0.074]	0.027 [0.016, 0.037)	0.052 [0.032, 0.072)	0.099 [0.064, 0.135)	0.185 (0.146, 0.224]
								
	Overall	**Total**	**0.051 (0.029, 0.073]**	**0.050 [0.029, 0.070]**	**0.025 [0.014, 0.035]**	**0.049 [0.029, 0.068)**	**0.093 [0.059, 0.127)**	**0.175 (0.137, 0.214)**
	SHWs	Urban	0.151 (0.067, 0.235]	0.189 (0.056, 0.323)	0.080 (0.033, 0.128]	0.172 (0.063, 0.282]	0.396 [0.139, 0.652]	0.301 (0.222, 0.381]
		Rural	0.071 (0.024, 0.118]	0.069 (0.025, 0.112]	0.034 [0.012, 0.056]	0.066 (0.026, 0.107)	0.127 [0.054, 0.200)	0.201 (0.136, 0.265]
**Dire-Dawa**								
	HOs	**Total**	**0.066 [0.015, 0.117)**	**0.074 [0.019, 0.129]**	**0.034 (0.009, 0.060]**	**0.072 [0.020, 0.123)**	**0.150 (0.053, 0.247]**	**0.199 (0.114, 0.295]**
		Urban	0.082 (0.024, 0.141)	0.090 [0.031, 0.149]	0.042 (0.014, 0.071)	0.086 (0.032, 0.140]	0.172 (0.075, 0.269]	0.220 [0.121, 0.320)
		Rural	0.026 (0.001, 0.052]	0.028 (0.002, 0.054]	0.014 (0.001, 0.026]	0.028 (0.002, 0.053]	0.056 [0.005, 0.108)	0.121 (0.033, 0.208]
	Nurses	**Total**	**0.057 [0.005, 0.108)**	**0.066 (0.008, 0.124]**	**0.030 [0.003, 0.057)**	**0.064 [0.009, 0.119)**	**0.141 [0.029, 0.252]**	**0.178 [0.085, 0.272)**
		Urban	0.060 (0.006, 0.113]	0.067 [0.009, 0.125)	0.031 (0.004, 0.058]	0.065 [0.010, 0.119]	0.136 [0.030, 0.242]	0.185 (0.083, 0.287)
		Rural	0.006 (0.001, 0.011)	0.006 (0.001, 0.011)	0.003 (0.000, 0.005]	0.006 [0.001, 0.011)	0.012 (0.002, 0.022)	0.056 [0.018, 0.095]
	Midwives	**Total**	**0.134 (0.028, 0.240)**	**0.152 [0.020, 0.283]**	**0.069 [0.013, 0.124]**	**0.141 (0.027, 0.255)**	**0.291 (0.067, 0.516)**	**0.276 (0.144, 0.409]**
		Urban	0.073 [-0.022, 0.168)	0.093 [-0.016, 0.201]	0.040 (-0.009, 0.089]	0.088 [-0.011, 0.188]	0.205 [0.007, 0.402)	0.186 [0.039, 0.333)
		Rural	0.039 (0.000, 0.079)	0.043 (0.003, 0.084)	0.020 (0.001, 0.040]	0.042 (0.003, 0.082)	0.088 [0.009, 0.167)	0.141 (0.030, 0.253)
						
	Overall	**Total**	0.063 (0.003, 0.122]	0.073 [0.005, 0.141]	0.033 [0.002, 0.064]	0.071 [0.007, 0.134)	0.156 (0.031, 0.280)	0.186 (0.085, 0.286]
	SHWs	Urban	0.060 (0.006, 0.114)	0.069 [0.011, 0.127)	0.032 [0.004, 0.059)	0.067 (0.012, 0.121]	0.143 (0.037, 0.249]	0.177 [0.071, 0.283)
		Rural	0.012 (0.000, 0.023)	0.012 (0.001, 0.023]	0.006 (0.000, 0.011]	0.012 (0.001, 0.023]	0.025 [0.002, 0.047]	0.076 (0.014, 0.138]

**HOs**: health officers; **SHWs**: skilled health workers

**Table 5:** Inequality indices for accessibility of SHWs in Afar during 2017.

SHWs		Theil Indices (95% CI)		Atkinson Indices (95% CI)			Gini Index (95% CI)
		TT at α = 1	TL at α = 0	AI at ε = 0.5	AI at ε = 1	AI at ε = 2	
**Health Officers**							
	**Total**	**0.281 [0.179, 0.382]**	**0.295 (0.197, 0.392]**	**0.136 (0.091, 0.180)**	**0.255 [0.182, 0.328]**	**0.427 (0.332, 0.523)**	**0.412 (0.341, 0.484)**
	NHA	0.238 (0.133, 0.344)	0.262 [0.151, 0.373]	0.118 [0.069, 0.167]	0.231 (0.144, 0.318)	0.414 [0.287, 0.541]	0.381 (0.289, 0.473)
Allowance	30%	0.230 (0.116, 0.345)	0.220 (0.102, 0.337]	0.108 [0.054, 0.161]	0.197 [0.098, 0.296]	0.318 (0.156, 0.479]	0.366 [0.254, 0.477]
	40%	0.245 (0.039, 0.451]	0.246 [0.027, 0.465)	0.117 [0.018, 0.217)	0.218 [0.033, 0.403)	0.358 (0.061, 0.655]	0.373 (0.152, 0.594]
Altitude	>1000	0.187 (-0.079, 0.453]	0.270 [-0.030, 0.571)	0.107 (-0.025, 0.239]	0.237 (-0.018, 0.491]	0.478 [0.032, 0.924]	0.268 (-0.040, 0.576]
(masl)	500–1000	0.230 [0.114, 0.346)	0.243 [0.123, 0.362]	0.112 (0.059, 0.166)	0.215 (0.121, 0.310]	0.381 [0.243, 0.519)	0.375 [0.276, 0.473]
	<500	0.231 (0.083, 0.380]	0.207 [0.065, 0.349]	0.105 [0.037, 0.173)	0.187 (0.066, 0.309)	0.295 (0.108, 0.481]	0.358 [0.211, 0.504]
**Nurses**							
	**Total**	**0.231 [0.125, 0.337]**	**0.258 [0.144, 0.371]**	**0.115 (0.067, 0.162]**	**0.227 (0.141, 0.314)**	**0.440 (0.294, 0.586]**	**0.372 [0.290, 0.454]**
	NHA	0.187 (0.079, 0.295]	0.196 [0.086, 0.305)	0.091 (0.042, 0.141)	0.178 (0.088, 0.268)	0.332 [0.184, 0.481)	0.332 [0.232, 0.432]
Allowance	30%	0.229 (0.060, 0.399)	0.293 [0.057, 0.530)	0.121 [0.033, 0.209]	0.254 [0.079, 0.429]	0.518 [0.220, 0.815)	0.369 (0.226, 0.513]
	40%	0.086 [0.012, 0.160)	0.084 [0.011, 0.157)	0.042 [0.006, 0.077]	0.081 [0.012, 0.149)	0.146 (0.024, 0.269)	0.225 [0.098, 0.352]
							
Altitude	>1000	0.075 (0.003, 0.148)	0.077 (0.004, 0.151)	0.038 (0.002, 0.073]	0.075 [0.005, 0.144]	0.142 [0.013, 0.271)	0.209 [0.069, 0.350)
(masl)	500–1000	0.196 [0.063, 0.328)	0.212 [0.076, 0.349)	0.097 (0.036, 0.158)	0.191 [0.081, 0.302)	0.368 (0.197, 0.539]	0.337 (0.217, 0.457)
	<500	0.243 (0.079, 0.407]	0.257 [0.076, 0.438]	0.117 (0.041, 0.194)	0.227 [0.085, 0.368]	0.426 (0.190, 0.662]	0.373 (0.235, 0.512)
**Midwives**							
	**Total**	**0.242 [0.155, 0.329]**	**0.303 [0.182, 0.424)**	**0.126 (0.082, 0.171)**	**0.261 [0.172, 0.351)**	**0.528 (0.372, 0.684)**	**0.385 [0.315, 0.455)**
Allowance	NHA	0.151 [0.090, 0.211]	0.157 [0.091, 0.222)	0.074 (0.045, 0.104)	0.145 (0.089, 0.201]	0.265 [0.168, 0.363)	0.310 (0.246, 0.374)
	30%	0.228 (0.072, 0.384)	0.290 [0.108, 0.471)	0.121 (0.046, 0.196]	0.251 (0.114, 0.389]	0.485 [0.276, 0.694)	0.366 [0.231, 0.501)
	40%	0.202 (0.020, 0.383]	0.225 (0.022, 0.428]	0.102 [0.013, 0.190]	0.201 (0.036, 0.367]	0.372 [0.107, 0.637]	0.339 [0.152, 0.526]
							
Altitude	>1000	0.029 (0.003, 0.056)	0.029 (0.003, 0.056)	0.015 [0.001, 0.028)	0.029 (0.003, 0.055)	0.055 [0.005, 0.105]	0.130 (0.043, 0.217]
(masl)	500–1000	0.156 [0.086, 0.227)	0.163 [0.090, 0.235]	0.077 (0.044, 0.111)	0.150 [0.088, 0.212]	0.274 [0.171, 0.376)	0.315 (0.242, 0.388]
	< 500	0.434 (0.182, 0.685)	0.485 (0.210, 0.760]	0.208 [0.098, 0.317]	0.384 (0.205, 0.564)	0.612 (0.390, 0.834)	0.503 [0.353, 0.654)
Overall SHWs							
	**Total**	**0.196 [0.118, 0.274]**	**0.215 (0.130, 0.301)**	**0.098 [0.061, 0.135)**	**0.194 (0.125, 0.263)**	**0.366 (0.251, 0.481]**	**0.347 [0.277, 0.417)**
	NHA	0.145 (0.064, 0.226)	0.155 [0.071, 0.238)	0.072 (0.034, 0.111)	0.143 (0.072, 0.215)	0.276 [0.156, 0.396)	0.295 [0.203, 0.386]
Allowance	30%	0.211 [0.089, 0.333]	0.245 [0.094, 0.396]	0.108 (0.047, 0.170)	0.217 [0.098, 0.337)	0.412 [0.213, 0.610]	0.358 [0.245, 0.470]
	40%	0.024 (0.007, 0.042]	0.025 (0.007, 0.043)	0.012 [0.003, 0.021]	0.024 (0.007, 0.042]	0.048 (0.014, 0.083]	0.121 [0.055, 0.186]

**masl**: metres above sea level; **NHA**: no hardship allowance; **SHWs**: skilled health workers

### Inequality changes

We observed statistically significant inequality changes of all distributions from 2015 to 2017 only in Tigray (Table 6). The average growth rate (AGR) of 0.339 in the allocation of GHE towards the disadvantaged populations resulted in 10.00 percentage points reductions (p < 0.001) in the overall inequality in the regional GHE. The expansion of the HCs towards the privileged populations (AGR = 0.042) resulted in 19.33 percentage points increase in the overall inequality in HCs in the region, which was insignificant (p = 0.062). The allocation of HOs (AGR = 0.480), nurses (AGR = 0.106), midwives (AGR = 0.169) and overall SHWs (AGR = 0.170) towards the disadvantaged populations resulted in 20.20 percentage points (p = 0.003), 16.52 percentage points (p = 0.005), 13.82 percentage points (p = 0.032) and 19.60 percentage points (p < 0.001) reductions in the overall inequality of each category of SHWs in Tigray respectively. The changes in inequalities for Dire-Dawa and Afar regions were statistically insignificant.

**Table 6:** Inequalities in GHE per capita, HCs per 15,000 inhabitants and SHWs per 10,000 inhabitants and inequality change between 2015 and 2017 in Ethiopia.

Indicator		GI, 95%CI		Inequality change (Δ)			
		Year 2015	Year 2017	AGR	Δ (95% CI)	% Δ	p-value
GHE per capita							
	Tigray	0.197 [0.120, 0.274]	0.181 (0.118, 0.244)	0.339	-0.019 [-0.028, -0.010]	-10.00	<0.001
	Dire-Dawa	0.157 [0.057, 0.258)	0.152 [0.064, 0.239]	0.214	-0.005 [-0.026, 0.015]	-3.50	0.603
	Afar	0.269 [0.217, 0.322)	0.268 [0.207, 0.329)	0.855	-0.001 (-0.028, 0.025)	-0.50	0.912
HC/15000 pop							
	Tigray	0.181 [0.138, 0.224)	0.216 (0.144, 0.289)	0.042	0.035 (-0.002, 0.071]	19.33	0.062
	Dire-Dawa	0.280 [0.169, 0.390]	0.280 [0.168, 0.391]	-0.053	-0.000 (-0.000, 0.000)	-0.00	0.856
	Afar	0.223 [0.174, 0.271]	0.237 [0.164, 0.311)	0.034	0.014 [-0.021, 0.050]	6.50	0.430
Health officer							
	Tigray	0.302 (0.244, 0.360)	0.246 [0.191, 0.301)	0.480	-0.062 [-0.102, -0.021]	-20.20	0.003
	Dire-Dawa	0.230 (0.138, 0.322]	0.199 (0.114, 0.295]	-0.155	-0.031 [-0.067, 0.006)	-13.30	0.100
	Afar	0.386 (0.303, 0.469]	0.412 [0.345, 0.479]	0.101	0.026 (-0.023, 0.075]	6.74	0.296
Nurses							
	Tigray	0.230 (0.186, 0.275)	0.193 (0.154, 0.231]	0.106	-0.038 (-0.065, -0.011]	-16.52	0.005
	Dire-Dawa	0.182 [0.085, 0.280)	0.178 [0.085, 0.272)	0.034	-0.004 [-0.053, 0.045]	-2.20	0.870
	Afar	0.352 (0.284, 0.420]	0.372 [0.288, 0.456)	0.057	0.020 (-0.031, 0.071)	5.70	0.436
Midwives							
	Tigray	0.217 [0.187, 0.246]	0.187 [0.150, 0.224]	0.169	- 0.030 [-0.061, -0.001]	-13.82	0.032
	Dire-Dawa	0.252 [0.145, 0.259]	0.276 (0.137, 0.416]	-0.017	0.024 [-0.022, 0.071)	9.60	0.307
	Afar	0.391 [0.303, 0.478)	0.385 (0.308, 0.462]	0.185	- 0.006 [-0.055, 0.044]	- 1.40	0.825
Overall SHWs							
	Tigray	0.217 (0.177, 0.248)	0.175 (0.137, 0.214)	0.170	-0.043 (-0.063, -0.022)	-19.60	<0.001
	Dire-Dawa	0.193 (0.091, 0.294]	0.186 (0.082, 0.289)	-0.006	-0.007 [-0.047, 0.033]	-3.60	0.735
	Afar	0.339 [0.281, 0.397)	0.347 [0.275, 0.419)	0.089	0.008 (-0.031, 0.047)	2.30	0.688

**AGR**: average growth rate; **GHE**: government health expenditure; **HC**: health centre

## Discussion

This study analysed district level densities of available PHC resources and the magnitude and trend of their inequalities across three regions in Ethiopia. Our findings revealed regional differences in the accessibility of healthcare resources. The variations in GHE per capita among the regions might be due to differences in institutional capacity, accountability [23], access to resources, i.e. finance, and other contextual factors. Although Tigray had the lowest GHE per capita, it showed significant reductions in the inequalities of the GHE per capita and the SHWs compared to the other two regions. These progressive changes in the allocations of the healthcare resources might be due to improvements in governance, accountability, commitment of the local administrations and public participation for enhancing the accessibility of PHC services [24]. The relatively higher GHE per capita in Afar and Dire-Dawa regions might indicate their high expenditures on hardship allowances. One study in Uganda also found more spending mainly for wages than for the expansion of health infrastructure [25].

Fair accessibility of well-staffed PHC facilities is a prerequisite to ensure the coverage of essential healthcare services, thereby leading to reach UHC and reductions in healthcare outcome inequalities [26,27]. Our findings indicated that the median density of HCs in the rural districts of Tigray and Dire-Dawa were about 1.46 (p = 0.001) and 2.21(p = 0.010) times higher than the median densities in the urban districts of the corresponding regions. However, there was no significant difference in the density of HCs between the NHA districts and districts with HA in the Afar region. Regardless of these regional differences, our study revealed fewer overall density of HCs than that reported from other studies in Africa and elsewhere [15,28]. A study in a rural district in Ethiopia reported a relationship between low accessibility of a health centre and the risk of mortality in under-five children [29].

Inaccessibility to healthcare has been reported as a common challenge, especially in the public health sector of under-resourced countries [30]. The median of HOs and midwives per HC in the Afar region were below the minimum threshold [31]. The inadequately staffed health centres, the scattered and nomadic-pastoralist way of life for the majority of the Afar people, and the harsh climate might have hindered the accessibility of healthcare services in the region [32]. Evidence from Chad also showed that accessible healthcare services to mobile pastoralists contributed to the achievement of 80% antenatal care contact coverage [33]. Besides, there is a positive association between population density and coverage of PHC services [34]. The relatively fair distribution of HCs and the inadequate staffing in Afar required attention to ensure the accessibility of PHC services. Another study in Africa reported a critical shortage of SHWs at PHC centres than at higher-level health facilities [35].

The densities of SHWs per health centre in Tigray and Dire-Dawa were in line with the minimum requirement of the Ethiopian Standard [31]. Our findings revealed compliance of the minimum threshold of three midwives per health centre in Tigray, while previous study in the same region reported 50% fulfillment [36]. This difference might be due to the progressive staffing of the health centres. However, in Afar, only the density of nurses was almost in line with the minimum standard. One district-based study in southwest Ethiopia found a difference in the density of SHWs per HC, and an association between higher density of SHWs and the efficiency of HCs [37]. Despite the compliance of each category of SHWs per HC with the national minimum threshold in Tigray and Dire-Dawa, the densities of SHWs per 10,000 inhabitants were too small, which may imply the limitations of the per facility staffing norms [38].

The median overall SHWs (7.539) and nurses (5.154) each per 10,000 inhabitants in the urban setting of Dire-Dawa were higher than the average SHWs (7.37) and nurses (2.32) reported from a study in urban settings in China [39]. However, the densities of the nurses in all three regions in our study were lower than the density reported from Angola. The study in Angola also shows a higher density of nurses in rural than in urban areas [40]. The median of midwives per 10,000 inhabitants in rural areas of Dire-Dawa were about twice (p = 0.016) that of the urban area. In Afar, the medians of HOs and overall SHWs each per 10,000 inhabitants in the NHA districts (Med. = 1.167 and Med. = 8.233) were respectively about three times (p = 0.039) and two times (p = 0.033) higher than the median densities in districts with HA (Med. = 0.428 HOs and Med. = 3.833 overall SHWs). The regional differences in the accessibility of SHWs identified in our study are consistent with the evidence reported from studies elsewhere [41,42].

The median overall SHWs per 10,000 inhabitants in Afar (5.250), Tigray (6.246) and Dire-Dawa (7.539) regions were 23.03%, 27.39%, and 33.07% respectively of the WHO minimum threshold, which is 22.8 SHWs to achieve the coverage of essential healthcare services [43]. These densities were also 11.80%, 14.04%, and 16.94% (for the three concerned regions) of the WHO minimum threshold of 44.5 SHWs to achieve the UHC and SDGs for health by 2030 [44]. We found extremely low densities of SHWs, which might indicate the level of efforts required to progress towards UHC [42]. Unless mechanisms are devised to increase the staffing and retain adequate SHWs, the densities of the SHWs remain far below the minimum threshold to achieve SDGs [45].

The low density of SHWs in the pastoralist Afar region could be related to multiple factors. Staffing the HCs with an adequate number of SHWs and locating them close to where the people live, particularly for the nomadic people who move a lot, have limited access to infrastructure, health facilities, safe drinking water, telephone networks, may contribute to reduction of inequalities in the accessibility of SHWs [32,46,47]. Afar is the region that is more likely to suffer from imbalances in the accessibility to PHC services. The relatively higher densities of SHWs in the NHA districts and the declining densities of SHWs along with declining average altitudes of the districts implies the harsher context for the SHWs to cope, which could be non-compensable by the existing rates of HA.

The low densities of SHWs plus the inequalities in their distributions found in our research, indicate the magnitude of existing challenges to achieve sustainable health development in Ethiopia. The Gini values for nurses during 2017 in Tigray (GI = 0.193) and Dire-Dawa (GI = 0.178) were lower, while in Afar (GI = 0.372) it was higher than that reported from Cameroon (GI = 0.308) [48]. The Gini values for nurses working in the urban settings in Tigray (GI = 0.179) and Dire-Dawa (GI = 0.185) were less than half of the Gini value for nurses (GI = 0.48) reported working in the urban settings in China [39]. In Afar region, the Gini value for nurses (GI = 0.509) was almost three times that of the Gini values for nurses in Tigray and Dire-Dawa regions and was higher than that reported from Fiji (G = 0.412) [49].

The increased GHE allocation towards the disadvantaged populations in Tigray resulted not only a significant overall GHE per capita inequality reduction (p < 0.001), but also associated with marked declines in SHWs inequalities in the region (p < 0.05). These achievements could be related to improvements in the overall governance and commitment of the local administrations [11,24]. However, the increasing tendency in HCs accessibility in favor of the advantaged populations (p = 0.062) might potentially increase the inequalities of the SHWs in Tigray. The inequality values for all indicators in Dire-Dawa were generally smaller than for the other two regions. The inequality changes between 2015 and 2017 for both Dire-Dawa and Afar regions remained insignificant. While allocating more SHWs could have contributed to the reduction in the inequalities [47], attracting and retaining the SHWs in such remote areas as Afar with a harsh climate and poor infrastructure is still a big challenge due to financial reasons as well as welfare and other conditions for employees. These issues call for a multi-sectoral development intervention to improve the availability and accessibility of other public services in the context.

## Strengths and limitations

To the best of our knowledge, this is the first study which investigates the population-based accessibility of PHC resources using district-level data of three regions representing the agrarian, pastoralist/semi-pastoralist, and urban regions in Ethiopia. The findings provided a clear picture, we envisage, about the densities of the PHC resources, and the magnitude and changes in inequalities. The comparisons between the urban and rural districts in Tigray and Dire-Dawa, and among the categories of districts based on context-specific criteria in Afar and the use of different inequality measures further provided evidence-informed policy recommendations to improve PHC services in the regions. Nonetheless, our study focuses mainly on the supply side of PHC system and cannot inform about the demand side, which might have influenced the accessibility of the PHC services in the contexts.

## Conclusions

Fair accessibility of PHC services can contribute to the achievement of SDGs for health. The HCs’ fair distributions across three regions in Ethiopia might imply the promising efforts of the local and regional administrations towards UHC. However, the low densities of SHWs, and the inequalities in their distributions, especially in the Afar region can hinder both the accessibility of the basic healthcare services and compromise the quality care, users’ satisfaction, and achieving health-related SDGs. Our findings indicate the need for further investigation to identify factors that contributed to the low densities of SHWs and the solutions to compensate hardship, so to attain a better density of SHWs, especially in the Afar region, and improve PHC services ultimately.

## Ethical considerations

### Ethics approval and consent

This study was approved by the Ethical Review Committee-Tehran University of Medical Sciences (ref. no. IR.TUMS.SPH.REC.1396.3213) and the Ethical Review Board at the Ethiopian Public Health Institute (ref. no. EPHI-IRB 041–2017). Data were collected after obtaining a formal letter of cooperation from the FMOH of Ethiopia and verbal informed consent from all responsible bodies in the study areas. There was no direct involvement of the individual health workers and the public in the data collection and analysis.

## Supporting information

S1 Dataset Minimum dataset of Tigray, Dire-Dawa and Afar regions in Ethiopia.xlsx. NB: The average annual government health expenditure (GHE) per capita is in Ethiopian Birr; the health centres (HCs) in each district are ranked by 15000 population; the health officers, nurses, midwives, and the summation of these professionals together are ranked each by 10000 population; and the average altitudes of the districts in Afar region are retrieved from different sources.
(XLSX)
